# Recurrent Duodenal Ulcer After Gastroduodenal Artery Embolization Due to Coil Migration Successfully Removed Endoscopically Resulting in Ulcer Healing

**DOI:** 10.7759/cureus.62972

**Published:** 2024-06-23

**Authors:** Isaac E Perry, Daniel Staursky, Mohammad Maysara Asfari, Kenneth J Vega

**Affiliations:** 1 Division of Gastroenterology and Hepatology, Augusta University Medical College of Georgia, Augusta, USA; 2 Department of Medicine, Augusta University Medical College of Georgia, Augusta, USA

**Keywords:** upper endoscopy, migration, coil, embolization, duodenal ulcer

## Abstract

Transarterial angiographic embolization using coils is an effective, common, and safe treatment for non-variceal upper gastrointestinal bleeding (UGIB) refractory to endoscopic therapy/management. Coil migration is a complication that can lead to rebleeding. Our patient experienced UGIB due to a recurring duodenal ulcer with coil protrusion following previous embolization for a bleeding duodenal ulcer that was not responsive to endoscopic therapy. The ulceration was successfully managed with endoscopic partial coil removal and medical therapy to achieve hemostasis and ulcer healing. Endoscopists should be aware of coil embolization complications and consider endoscopic removal in the appropriate clinical setting.

## Introduction

Non-variceal upper gastrointestinal bleeding (UGIB) is among the most common gastrointestinal illnesses leading to hospitalization [1]. *Helicobacter pylori* infection has historically been a primary cause of peptic ulceration that frequently leads to UGIB. Mainstays of treatment for UGIB include fluid resuscitation and blood transfusions, proton pump inhibitors, and endoscopic intervention [2]. Surgical intervention and transarterial angiographic embolization (TAE) can be utilized for persistent bleeding refractory to endoscopy [3]. TAE utilizing coil embolization has been shown to be successful and offers advantages compared to surgery. Coil migration is a rare and at times dangerous complication [4]. We present a rare case of UGIB caused by coil migration and protrusion in a recurring duodenal ulcer subsequent to coil embolization to manage a previously bleeding duodenal ulcer resistant to endoscopic therapy.

## Case presentation

**Figure 1:**
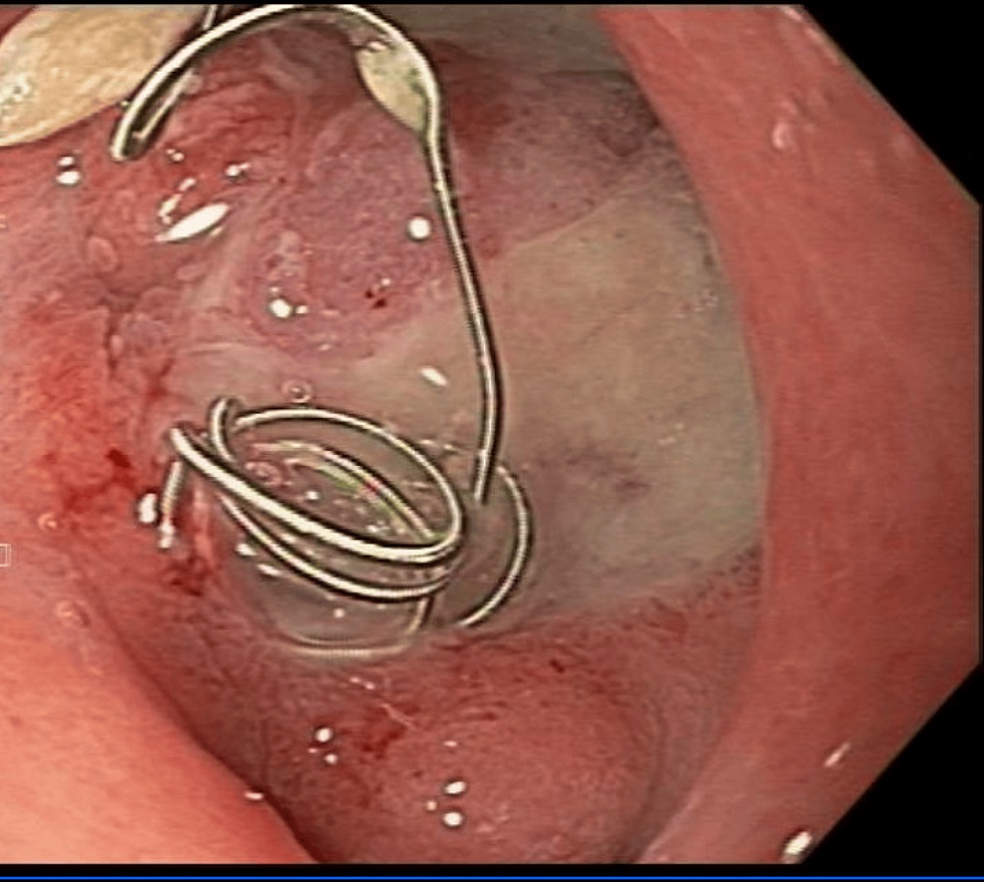
Forrest III duodenal ulceration with coil protrusion.

A 55-year-old male with a history of severe gastrointestinal bleeding from an *H. pylori*-induced duodenal ulcer which required gastroduodenal artery coil embolization for control of hemorrhage was admitted to our facility for substernal chest pain, melena, and a 2 g drop in hemoglobin from his baseline. The patient reported multiple episodes of melena in the two days before the presentation. Pertinent history also included *H. pylori* infection that was eradicated and confirmed via repeat biopsies during a previous esophagogastroduodenoscopy. Urgent endoscopy revealed two separate 1 cm Forrest III duodenal ulcers with coil protrusion in one of the ulcers (Figure 1).

**Figure 2:**
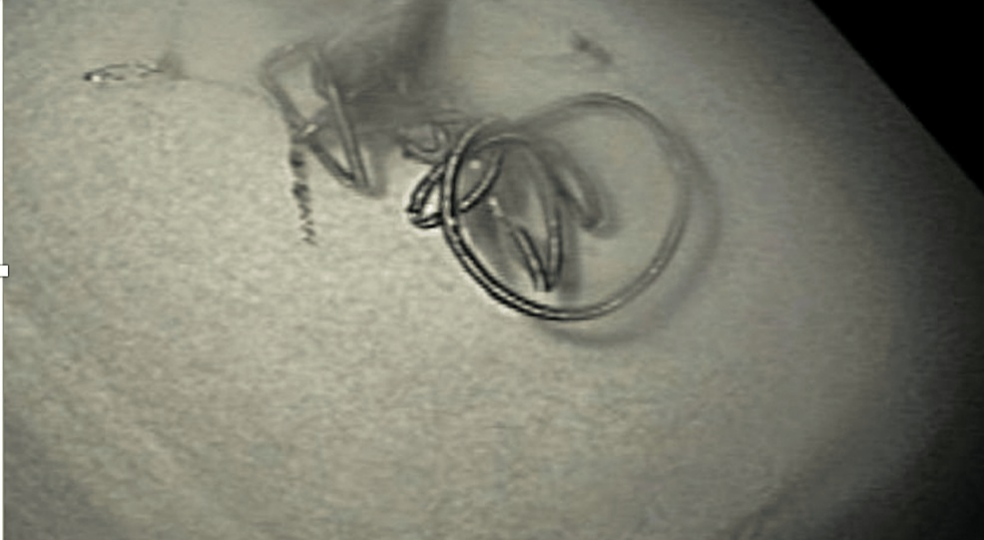
Protruding embolization coil post-removal.

The visible coil was cut with endoscopy scissors and extracted using rat tooth forceps without recurrence of melena (Figure 2).

Repeat biopsies taken did not reveal *H. pylori* infection. Following intervention, there was no further evidence of bleeding and the patient was discharged without complication on a proton pump inhibitor taken twice daily. A repeat endoscopy three months later revealed a deformed pylorus and residual ulcer which had healed by approximately 50%.

## Discussion

Non-variceal UGIB remains a common diagnosis that carries a heavy burden in terms of emergency room visits, hospitalizations, and costs. Peptic ulcer disease accounts for a significant amount of acute non-variceal UGIB [5]. Total hospital costs in the United States for peptic ulcer bleeding are estimated at $750 million [1]. While endoscopy remains the first line for interventions regarding UGIB, surgery and TAE remain options for bleeding refractory to endoscopy [5]. TAE is an effective therapy and can be an alternative to surgery in refractory non-variceal UGIB. Technical and clinical success rates for duodenal ulcer bleeding refractory to endoscopy treated with TAE are shown to be as high as 100% and 83%, respectively [6-8]. A lower 30-day mortality benefit in patients with endoscopy refractory UGIB has been seen with TAE (3%) compared with surgery (14%). TAE has also been shown to carry lower rates of post-procedural complications in comparison to surgical intervention [3,9]. While rare, TAE is not without risks. Complications include hematoma, arterial dissection, contrast complications, and coil migration [10]. Coil migration can occur intraprocedurally or weeks to even years after placement [11].

Our patient’s presentation was unique in that a previously placed coil for duodenal ulcer bleeding migrated and protruded through a duodenal ulcer resulting in recurrent UGIB. Management of coil migration into an ulcer remains unclear. Several case reports describe conservative management without retrieval of the coil. The majority of these cases involved migration of the coil into the small bowel lumen and resulted in coil passage [12]. Other cases were managed by open surgical retrieval [13]. A single case involving duodenal bulb ulcers complicated by coil protrusion was conservatively managed and demonstrated ulcer healing without describing coil passage [14].

## Conclusions

We report a rare case of a life-threatening recurrent UGIB in a duodenal ulcer caused by coil migration and successfully treated with endoscopic visible coil extraction. Endoscopic coil extraction as a method of treatment can be considered as an option, especially in patients who are at poor surgical risk.
